# Low-density lipoprotein receptor (LDLR) regulates NLRP3-mediated neuronal pyroptosis following cerebral ischemia/reperfusion injury

**DOI:** 10.1186/s12974-020-01988-x

**Published:** 2020-11-05

**Authors:** Rui Sun, Mengna Peng, Pengfei Xu, Feihong Huang, Yi Xie, Juanji Li, Ye Hong, Hongquan Guo, Qian Liu, Wusheng Zhu

**Affiliations:** 1 Department of Neurology, Jinling Clinical College of Nanjing Medical University, 305 East Zhongshan Road, Nanjing, 210002 Jiangsu Province China; 2 Department of Neurology, Shanghai Changhai Hospital, Second Military Medical University/Naval Medical University, Shanghai, 200433 China; 3 Department of Neurology, Jinling Hospital, Medical School of Nanjing University, Nanjing, 210002 China; 4 Stroke Center & Department of Neurology, The First Affiliated Hospital of USTC, Division of Life Sciences and Medicine, University of Science and Technology of China, Hefei, 230036 Anhui China; 5 Department of Neurology, Jinling Hospital, the First School of Clinical Medicine, Southern Medical University, Nanjing, 210002 China

**Keywords:** Low-density lipoprotein receptor (LDLR), Inflammasome, Pyroptosis, Neuroinflammation, Ischemia/reperfusion

## Abstract

**Background:**

Inflammatory response has been recognized as a pivotal pathophysiological process during cerebral ischemic stroke. NLRP3 inflammasome, involved in the regulation of inflammatory cascade, can simultaneously lead to GSDMD-executed pyroptosis in cerebral ischemia. Low-density lipoprotein receptor (LDLR), responsible for cholesterol uptake, was noted to exert potential anti-inflammatory bioactivities. Nevertheless, the role of LDLR in neuroinflammation mobilized by cerebral ischemia/reperfusion (I/R) has not been investigated.

**Methods:**

Ischemic stroke mice model was accomplished by middle cerebral artery occlusion. Oxygen-glucose deprivation was employed after primary cortical neuron was extracted and cultured. A pharmacological inhibitor of NLRP3 (CY-09) was administered to suppress NLPR3 activation. Histological and biochemical analysis were performed to assess the neuronal death both in vitro and in vivo. In addition, neurological deficits and behavioral deterioration were evaluated in mice.

**Results:**

The expression of LDLR was downregulated following cerebral I/R injury. Genetic knockout of *Ldlr* enhanced caspase-1-dependent cleavage of GSDMD and resulted in severe neuronal pyroptosis. LDLR deficiency contributed to excessive NLRP3-mediated maturation and release of IL-1β and IL-18 under in vitro and in vivo ischemic conditions. These influences ultimately led to aggravated neurological deficits and long-term cognitive dysfunction. Blockade of NLRP3 substantially retarded neuronal pyroptosis in *Ldlr*^−/−^ mice and cultured *Ldlr*^−/−^ neuron after experimental stroke.

**Conclusions:**

These results demonstrated that LDLR modulates NLRP3-mediated neuronal pyroptosis and neuroinflammation following ischemic stroke. Our findings characterize a novel role for LDLR as a potential therapeutic target in neuroinflammatory responses to acute cerebral ischemic injury.

## Highlights

1. LDLR expression is downregulated following ischemic stroke.
2. *Ldlr* knockout facilitates cerebral infarct volume enlargement and aggravates neurological deficits.
3. LDLR could suppress neuronal pyroptosis by inhibiting NLRP3 inflammasome activation.
4. Inhibition of NLRP3 protects against pyroptotic neuronal death in *Ldlr*^−/−^ mice after ischemia.

## Introduction

Ischemic stroke is a common vascular disease caused by an abrupt reduction or obstruction of cerebral blood flow [1–3]. It is one of the well-known leading causes of global death and permanent disability [4]. Post-ischemic sterile inflammation has both protective and deleterious effects on disease progression [5], among which the molecular mechanisms of neuronal inflammatory injury following cerebral ischemia are complex and remain to be fully understood.

In the central nervous system (CNS), acute cerebral ischemia/reperfusion (I/R) can trigger neuroinflammation as an essential pathophysiological process to activate the innate immune response and then a series of inflammatory cascades [6]. The initiation of the neuronal immune response involves the assembly of the inflammasome complexes. Inflammasomes are cytosolic multiprotein signaling platforms that consist of sensors such as pattern recognition receptors (PPRs) that defend against infection and damage signals [7]. Inflammasome assembly recruits pro-caspase-1 and then process it into the mature form as caspase-1 [8]. Active caspase-1 subsequently mediates the cleavage of gasdermin D (GSDMD) [9]. The cleavage of GSDMD releases an N-terminal fragment to bind to phosphatidylserine and cardiolipin on the plasma membrane and then exhibits the pore-forming activity, eliciting a form of lytic cell death known as pyroptosis [10, 11]. Pyroptosis is a type of programmed cell death characterized by DNA fragmentation, rapid plasma membrane rupture, cell swelling, and release of proinflammatory cellular contents [12, 13]. It has been shown that pyroptosis could be triggered by ischemic stroke and that GSDMD could serve as a key executioner of caspase-1-mediated pyroptosis during cerebral I/R injury [10].

Low-density lipoprotein receptor (LDLR), a membrane-spanning glycoprotein mediating the transport and metabolism of cholesterol-containing lipoprotein, is broadly expressed on multiple cell types in various tissues [14]. In the CNS, LDLR is mainly located on the neurons, astrocytes, and oligodendrocytes [15]. LDLR plays an important role in regulating the homeostasis of the bloodstream and intracellular cholesterol [16]. Notably, except for those canonical functions, it has been reported that an increase in LDLR expression participated in restricting the deleterious proinflammatory signals of pathogens and improving prognosis among patients in sepsis and septic shock [17]. However, the precise role of LDLR in the neuronal inflammatory response following cerebral I/R injury remains largely unknown.

Thus, in this study, using mouse middle cerebral artery occlusion (MCAO) model and neuronal oxygen-glucose deprivation (OGD) model, we aimed to investigate the cellular location and temporal expression of LDLR and determine whether LDLR could orchestrate post-stroke neuronal inflammatory response and pyroptosis.

## Materials and methods

### Animals

Wild-type (WT) male C57BL/6 mice were purchased from the Model Animal Research Centre of Nanjing University (Nanjing, China). C57BL/6 *Ldlr* knockout mice were purchased from the Nanjing Biomedical Research Institute of Nanjing University (certificate no. T001464). Mice were housed in a temperature- and humidity-controlled environment (temperature 23 ± 2 °C; humidity 55–60%) with a 12-h light/12-h dark cycle and free access to chow and water. All experimental protocols were performed in accordance with the National Institutes of Health “Guide for the Care and Use of Laboratory Animals” (NIH Publications no. 8023, revised 2011) and approved by the Experimental Animal Ethics Committee of Jinling Hospital. At least three animals were analyzed for each data point.

### Focal cerebral ischemia

A total of 180 male C57BL/6 mice weighing 20–25 g (8–12 weeks old) were used in this study. The focal cerebral ischemia was accomplished by the endovascular MCAO model according to previous methods [7, 18]. In brief, animals were anesthetized with 2% isoflurane in O_2_, and the right common carotid artery, external carotid artery (ECA), and internal carotid artery (ICA) were carefully isolated through a midline cervical incision. Next, a silicon-coated monofilament (diameter 0.16 ± 0.02 mm) was inserted through the ECA and advanced into the ICA to occlude the origin of the middle cerebral artery (MCA). After ischemia for 90 min, the suture was withdrawn to allow reperfusion. The sham operation was conducted with the same procedure, except for MCA occlusion. The body temperature of the mice was maintained at 37.0 °C ± 0.5 °C by a heating pad until the skin incision was sutured. Altogether, 20–30% of animals were excluded because of hemorrhage, death, or failure to cause focal ischemia during surgery or after surgery.

### Oxygen-glucose deprivation and reoxygenation (OGD/R)

The primary cortical neurons were extracted from the cortex of C57BL/6 mouse embryos (E14) as previously described [19]. The meninges and blood vessels were removed, followed by digestion with 0.125% trypsin for 15 min. The cells were suspended in DMEM/10% fetal bovine serum and then seeded into flasks. After cell adherence, the medium was substituted with fresh neurobasal medium mixed with 2% B27 and 1% glutamax, and the medium was changed every three days. The cells were cultured for 5–6 days before the OGD experiment.

For OGD, neurons were transferred to glucose-free and serum-free DMEM and then incubated in an oxygen-free chamber aerated with 5% CO_2_ and 95% N_2_ at 37 °C. After 2 h, the neurons were returned to normal culture conditions for reoxygenation.

### Drug administration

A single dose of NLRP3 inhibitor CY-09 (40 mg·kg^−1^, i.p., Selleckchem, USA) was administered 1 h before MCAO surgery as described previously [20]. In cultured neurons, CY-09 (10 μM) was pre-incubated for 0.5 h before OGD. Then, neurons were incubated with CY-09 (10 μM) for 24 h after reoxygenation. Vehicle animals were treated with equivalent normal saline (i.p.) for comparison.

### Cerebral infarct volume and cerebral water content

At 24 h after reperfusion, the mice were anesthetized, and their brains were quickly removed. Then, 2,3,5-triphenyltetrazolium chloride (TTC, Sigma) staining was employed to measure brain infarct volume. Mouse brains were cut into 1-mm sections and stained with TTC solution for 15 min at 37 °C before being fixed with 4% paraformaldehyde (PFA) at 4 °C overnight. The relative infarct volume was calculated as reported previously [21]. The brain water content was measured with the wet-dry method [22]. Each hemisphere was weighed after removal and then weighed again at 105 °C overnight. The percentage of water content was calculated as [(wet weight − dry weight)/wet weight] × 100%.

### Evaluation of neurological deficits and behavioral analysis

Neurological deficits of the experimental mice were assessed with the modified neurologic severity score (mNSS) 24 h after reperfusion, as described [23]. The mNSS scoring system consists of four tests: sensory tests, motor tests, beam balance tests, and reflexes absent and abnormal movements. The mNSS is graded a scale of 0–18 points in which higher score indicates more severe neurological deficits. One point is recorded for the failure to perform the task or lack of a reflex. The score of 13–18 indicates severe injury while that of 1–6 indicates mild injury.

Spatial learning and memory were investigated with the Morris Water Maze (MWM) test on days 22–28 after reperfusion [24]. A blind test was performed prior to the experimental task on day 22 to exclude blind mice. In the place navigation test, animals were trained to find the platform in four trials for 5 days. Each trial lasted until the animal found the platform in 60 s. If the animal failed to reach the platform within 60 s, it would be guided there and rested for 10 s before the next experiment. In the spatial probe test on day 28, the platform was removed, and each animal was placed to swim freely for 60 s. The time spent in the target quadrant and the number of platform crossings were recorded and analyzed by the ANY-maze video tracking software (Stoelting, USA).

### Cell viability

Twenty-four hours after OGD, neuronal cell viability was detected with the Cell Counting Kit-8 (CCK-8) (Dojindo, Japan) according to the manufacturer’s instructions. The absorbance of each well was obtained at 450 nm. Cell viability was calculated by (experimental group absorbance value/control group absorbance value) × 100%.

Meanwhile, cell death was determined with a propidium iodide (PI)/Hoechst 33342 assay kit (Thermo Fisher Scientific, USA). The percentage of propidium iodide-positive neurons (red) compared with the total Hoechst-stained neurons (blue) was used to assess cell death. For each sample, four randomly selected areas were counted, and the average value was calculated.

### Fluoro-jade C (FJC) staining

Degenerated neurons were detected by FJC (Millipore, USA) as previously described [25]. Frozen slides were sequentially immersed in 1% sodium hydroxide solution, 70% ethanol, and 0.06% potassium permanganate solution. Then, the sections were then incubated with 0.0001% solution of FJC. Quantified analysis of FJC-positive neurons was performed with Image J software.

### Immunofluorescence

Anesthetized mice were successively perfused intracardially with 0.9% sodium chloride and 4% paraformaldehyde (PFA). Mouse brains were soaked in 4% PFA for 12 h and then dehydrated by sucrose solutions with an ascending concentration gradient of 10, 20, and 30% at 4 °C. After fixation in optimal cutting temperature compound (Sakura Finetek, USA), the brains were sliced into 15-μm sections. Brain sections and neuron coverslips were fixed with 4% PFA for 20 min and then permeabilized with blocking buffer comprising 5% goat serum, 1% bovine serum albumin (BSA) and 0.3% Triton X-100 at room temperature for 1 h. The samples were incubated overnight with primary antibodies against LDLR (1:200, Santa Cruz Biotechnology, USA), NeuN (1:500, Abcam, UK), GFAP (1:500, Abcam, UK), Iba-1 (1:100, Abcam, UK), NLRP3 (1:200, Abcam, UK), ASC (1:200, Santa Cruz Biotechnology, USA), Caspase-1 (1:200, Santa Cruz Biotechnology, USA), and Gasdermin D (1:200, Santa Cruz Biotechnology, USA) at 4 °C, followed by incubation with appropriate Alexa Fluor-488/594-conjugated secondary antibodies (Jackson ImmunoResearch) and DAPI. Immunofluorescence images were captured with a microscope (Olympus MX51, Japan). The positive signals were analyzed using ImageJ software.

### Real-time quantitative PCR

Total RNA was extracted from cerebral tissues or cultured neurons utilizing TRIzol Reagent (Sigma, USA), and cDNA was reverse transcribed with a PrimeScript RT reagent kit (Thermo Fisher Scientific, USA). Real-time PCR was implemented in a Stratagene Mx3000P QPCR system (Agilent Technologies, USA) using a reaction system (UItraSYBR Mixture (ComWin Biotech, China), specific primers, diluted cDNA). The levels of glyceraldehyde-3-phosphate dehydrogenase (GAPDH) were set as the internal reference to assess the expression of target genes. The primer pairs are listed in Table 1.

**Table 1:** Real-time PCR primers used in this study

Primer name	Primer sequence	
NLRP3	Forward	ATGCTGCTTCGACATCTCCT
	Reverse	AACCAATGCGAGATCCTGAC
Pro-IL-1β	Forward	CAGGCAGGCAGTATCACTCA
	Reverse	AGGCCACAGGTATTTTGTCG
Pro-IL-18	Forward	GACTCTTGCGTCAACTTCAAGG
	Reverse	CAGGCTGTCTTTTGTCAACGA
GAPDH	Forward	AAGAAGGTGGTGAAGCAGGC
	Reverse	TCCACCACCCAGTTGCTGTA

### ELISA assay for inflammatory cytokines

IL-1β and IL-18 in cerebral ischemic penumbra and neuron culture supernatants were detected and quantified by ELISA kits (Abcam, UK) following the manufacturer’s instructions. Briefly, the supernatant of brain tissue homogenate or neuronal medium was added to 96-well plates coated with the indicated antibodies. After the reaction between the enzyme and substrate, the absorbances of the sample were assessed at 450 nm using a microplate reader (Thermo Fisher Scientific, USA).

### Electron microscopy

Tissues (1 × 1 × 1 mm) dissected from the cerebral ischemic penumbra were successively fixed in 2.5% glutaraldehyde and 1% osmium tetroxide and then cut into 50–60 nm slices after dehydration and insertion. Samples were observed and scanned using an H7500 Transmission Electron Microscope (Hitachi, Japan).

### CHOD-PAP method

Cholesterol levels in fresh venous serum and various brain regions were abstracted and detected using a Cholesterol Kit (Biosino Bio-technology, China) following the manufacturer’s instructions.

### Immunoblotting analysis

Immunoblotting analysis was performed as reported previously [19, 26]. Protein samples from brain tissues and cultured neurons were extracted using RIPA lysis buffer (Cell Signaling Technology, USA). The concentrations of these samples were detected with a BCA assay (Generay Biotechnology, China). Protein samples (20 μg for cells, 30 μg for tissues) were subjected to 8–12% sodium dodecyl sulfate-polyacrylamide gel electrophoresis (SDS-PAGE) and then transferred to polyvinylidene difluoride (PVDF) membranes (Millipore, USA). The membranes were probed overnight at 4 °C with primary antibodies against LDLR (1:200, Abcam, UK), NLRP3 (1:500, Abcam, UK), ASC (1:500, Santa Cruz Biotechnology, USA), Caspase-1 p10 (1:500, Santa Cruz Biotechnology, USA), GSDMD (1:500, Santa Cruz Biotechnology, USA), IL-1β (1:500, Santa Cruz Biotechnology, USA), IL-18 (1:500, Abcam, UK), nuclear factor kappa B (NF-κB) p65 (1:1000, Cell Signaling Technology, USA), phosphorylated-NF-κB p65 (1:1000; Abcam, UK), and β-actin (1:3000, Cell Signaling Technology, USA). The membranes were then incubated with horseradish peroxidase (HRP)-conjugated secondary antibody for 1 h at room temperature. Protein signals were detected by enhanced chemiluminescence solution (ECL, Millipore, USA). Quantitative analysis of protein bands was conducted using ImageJ software. β-actin served as the internal control.

### Statistical analysis

Experimental data were analyzed by SPSS 22.0 software (SPSS, Chicago, IL, USA). All values are expressed as the mean ± SEM. Escape latency and swimming path length in the MWM test were analyzed by two-way repeated-measures ANOVA followed by the least-significant-difference (LSD) post hoc test to determine differences between groups. Other results were analyzed using an independent sample *t* test for comparing two groups and one-way ANOVA for comparing multiple groups followed by LSD post hoc test. Statistical significance was taken as *P* < 0.05.

## Results

### The expression of neuronal LDLR is decreased following cerebral I/R

**Fig. 1:**
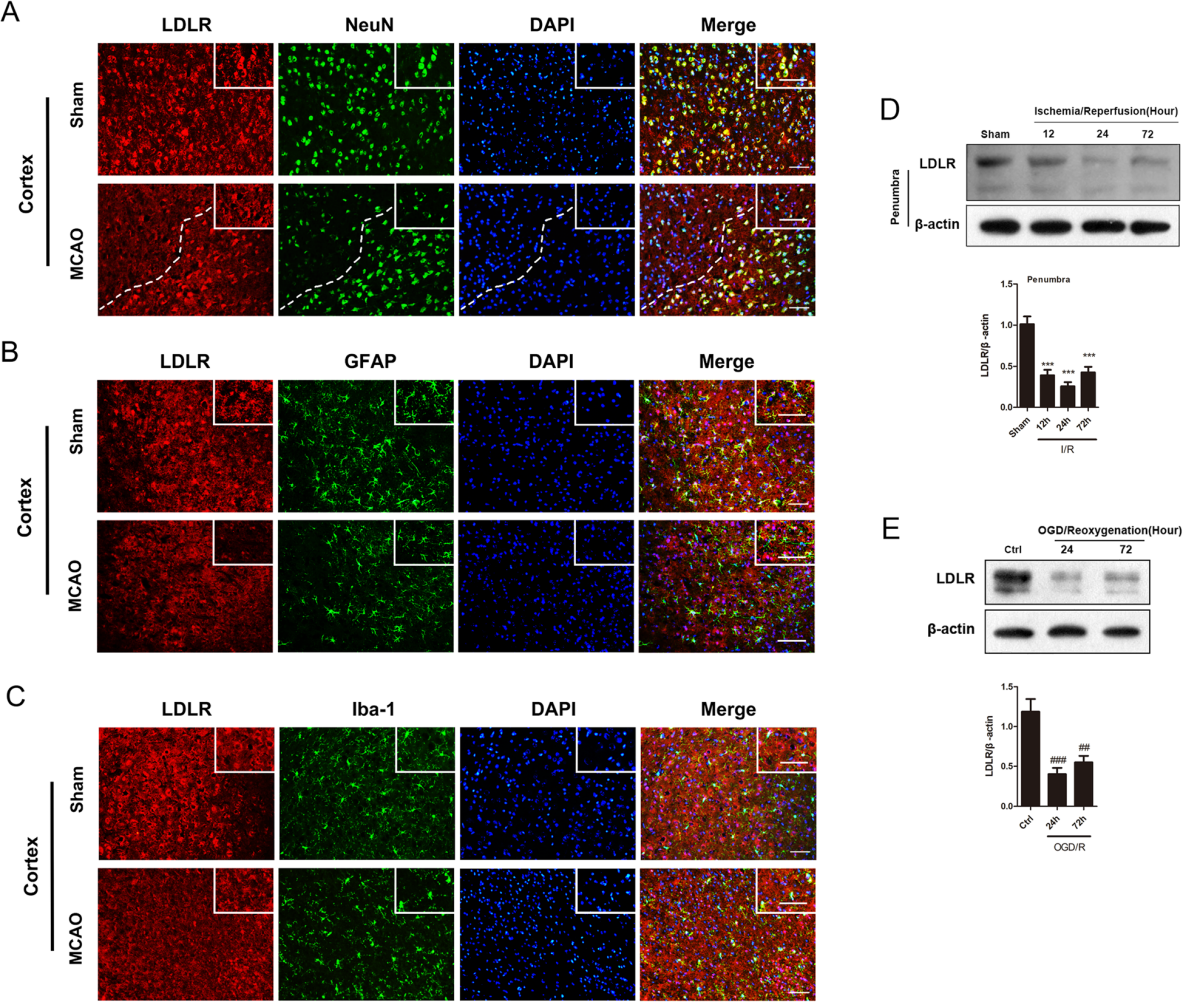
The expression of neuronal LDLR is decreased following cerebral I/R. **a** Representative immunofluorescence images of LDLR were co-stained with NeuN (neuron marker). LDLR positive neurons declined in ischemic penumbra. Insets show a higher magnification view. *n* = 3 in each group. **b**, **c** Double immunostaining of LDLR with GFAP (astrocyte marker) or Iba-1 (microglia marker) in the cortex of sham and MCAO mice revealed a modest co-localization. Insets show a higher magnification view. *n* = 3 for each group. **d** The expression and time course of LDLR in ischemic penumbra, *n* = 7 for all groups. **e** Immunoblotting analysis and quantification of LDLR in OGD-treated neurons, *n* = 5. Data are shown as mean ± SEM. ^***^P < 0.001 versus sham mice. ^##^*P* < 0.01, ^###^*P* < 0.001 versus control neurons. Ctrl, control. Scale bar = 20 μm

Immunofluorescence results indicated that LDLR was mainly detected in the cortex and corpus callosum of mice, with relatively fewer present in the hippocampus (Fig. 1a, S1A, S1B). In the cerebral cortex, LDLR signals were chiefly observed in the membrane and cytoplasm of neurons, rather than in the GFAP-positive astrocytes or Iba1-positive microglia (Fig. 1a–c). These results indicated that LDLR was mainly expressed in neurons located in the cortical area.

In MCAO mice, we observed weaker immunostaining intensity of neuronal LDLR in the peri-infarct regions than in sham-operated mice (Fig. 1a). Next, we assessed the temporal profile of LDLR protein expression in ischemic penumbra post-stroke. The western blotting results indicated that LDLR level was decreased after reperfusion and showed a robust decline at 24 h (Fig. 1d, compared to sham 12 h group, *P* < 0.001 for reperfusion 12 h; *P* < 0.001 for reperfusion 24 h; *P* < 0.001 for reperfusion 72 h). Furthermore, the expression of LDLR in cultured neurons also declined 24 h after reoxygenation (Fig. 1e, compared to the control 24 h group, *P* < 0.001 for reoxygenation 24 h; *P* = 0.002 for reoxygenation 72 h).

### *Ldlr* knockout aggravates post-stroke neurological deficits, infarct progression, and brain edema

**Fig. 2:**
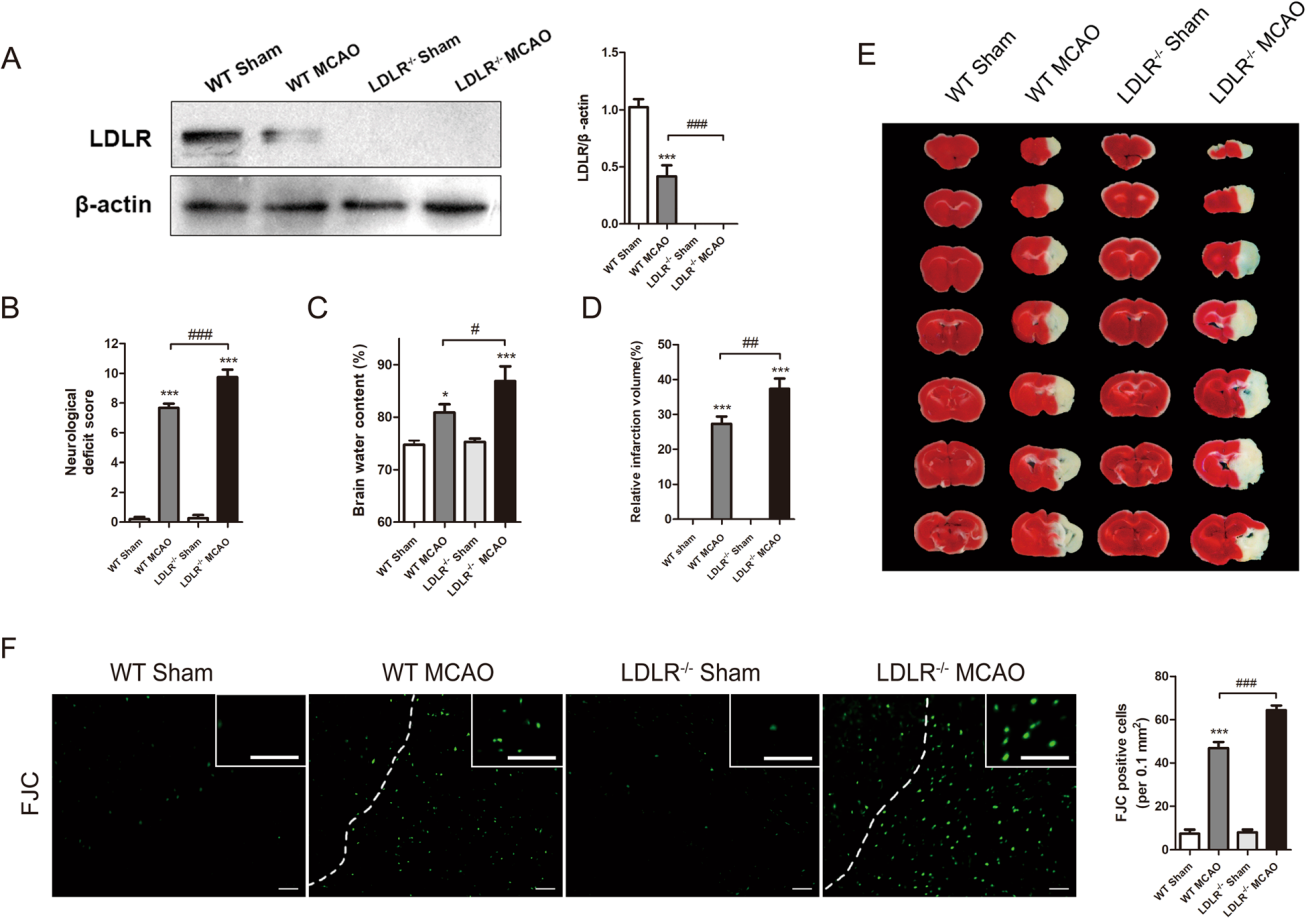
*Ldlr* knockout aggravates post-stroke neurological deficits, brain edema, infarct progression, and neuronal degeneration. **a** Immunoblots and quantitative analysis of LDLR, *n* = 4 in each group. **b** Neurological scores assessment of mice in each group, *n* = 15. **c** Quantitative analysis of brain edema, *n* = 4. **d**, **e** Representative image of brain slices by TTC staining in each group 1 day after reperfusion and the quantitative analysis of infarct volume, *n* = 7. **f** FJC staining showed that the density of FJC-positive neurons was increased after MCAO and was further aggrandized by *Ldlr* deletion, *n* = 4. Insets show a higher magnification view. Scale bar = 50 μm. All results are displayed as mean ± SEM. *P < 0.05, ***P < 0.001 versus WT sham mice; ^#^*P* < 0.05, ^##^*P* < 0.01, ^###^*P* < 0.001 versus WT MCAO mice

We further utilized *Ldlr* knockout mice to examine the physical function of LDLR in ischemic stroke. Western blotting analysis confirmed the complete knockout of LDLR protein in *Ldlr*^−/−^ mice (Fig. 2a, compared to WT sham group, *P* < 0.001 for *Ldlr*^−/−^ sham group). Due to the significant change of LDLR expression at 24 h after reperfusion, we chose 24 h as the detecting timepoint after MCAO treatment.

The mNSS assessment showed that MCAO surgery significantly destroyed sensorimotor function, as the neurological deficit score climbed to 7.67 ± 0.29 (Fig. 2b, compared to WT sham group, *P* < 0.001). In MCAO-treated *Ldlr*^−/−^ mice, neurological function was further exacerbated with a score of 9.73 ± 0.51 (Fig. 2b, compared to the WT MCAO group, *P* < 0.001). Regarding the brain water content, a similar tendency was observed in *Ldlr*^−/−^ MCAO mice where it changed from 80.89% ± 1.55% to 86.88% ± 2.82% (Fig. 2c, compared to WT MCAO group, *P* = 0.028). As demonstrated in Fig. 2d and e, the percentage of infarct volume was markedly enlarged to 37.34% ± 2.96% by genetic ablation of *Ldlr* following MCAO, as detected by TTC staining (compared to WT MCAO group, *P* = 0.001). We then used FJC staining to test whether LDLR could affect the neuronal degeneration after ischemic stroke. The images displayed that FJC-positive neurons after MCAO were significantly increased by inhibition of LDLR (Fig. 2f, compared to WT MCAO group, p < 0.001). These results suggested that *Ldlr* knockout aggravates early brain injury after ischemic stroke.

### *Ldlr* knockout exacerbates long-term cognitive deterioration

**Fig. 3:**
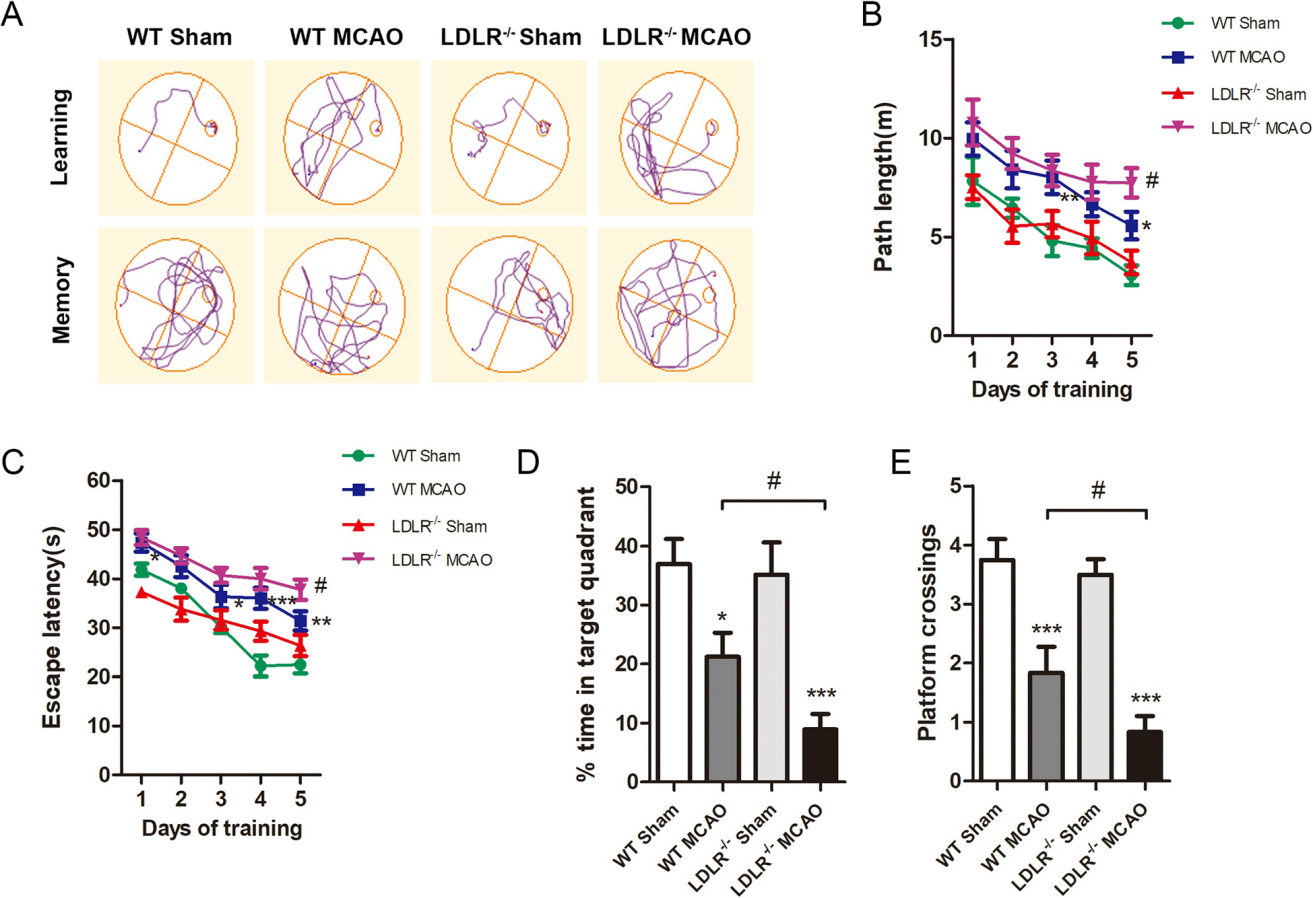
*Ldlr* knockout exacerbates long-term cognitive deterioration after stroke. **a** Representative swim path trace images of mice in hidden platform test (learning) and probe phase (memory). **b**, **c** The swim path length and escape latency were respectively detected at day 23–27 after reperfusion. **d**, **e** The percentage of time spent in the target quadrant and platform crossings were recorded at day 28 after reperfusion. Data are shown as mean ± SEM; *n* = 12 for all groups. *P < 0.05, **P < 0.01, ***P < 0.001 versus WT sham mice; ^#^*P* < 0.05 versus WT MCAO mice

To detect long-term spatial learning and memory function, we next performed MWM tests. The neurobehavioral capacities of *Ldlr*^−/−^ and their WT littermates both deteriorated in response to cerebral I/R, and compared with WT MCAO mice, *Ldlr*^−/−^ MCAO mice swam a longer distance and took more time to reach the platform on the 5th training day (Fig. 3a–c, compared with WT MCAO group, *P* = 0.045 for path length; *P* = 0.028 for escape latency). In the probe phase, no significant differences were observed in the WT sham and *Ldlr*^−/−^ sham groups. Both the time spent in the target quadrant and platform crossovers of WT MCAO mice were identically inhibited in the place navigation test (Fig. 3d, e, compared with WT sham group, *P* = 0.012). Simultaneously, LDLR deficiency conspicuously decreased the time spent in the target quadrant and platform crossovers (Fig. 3d, e, compared with the WT MCAO group, *P* = 0.046 and *P* = 0.043, respectively). Taken together, the loss of LDLR may have substantial detrimental effects on long-term behavioral outcomes following cerebral ischemic stroke.

### LDLR deficiency promotes neuronal pyroptosis post-stroke

**Fig. 4:**
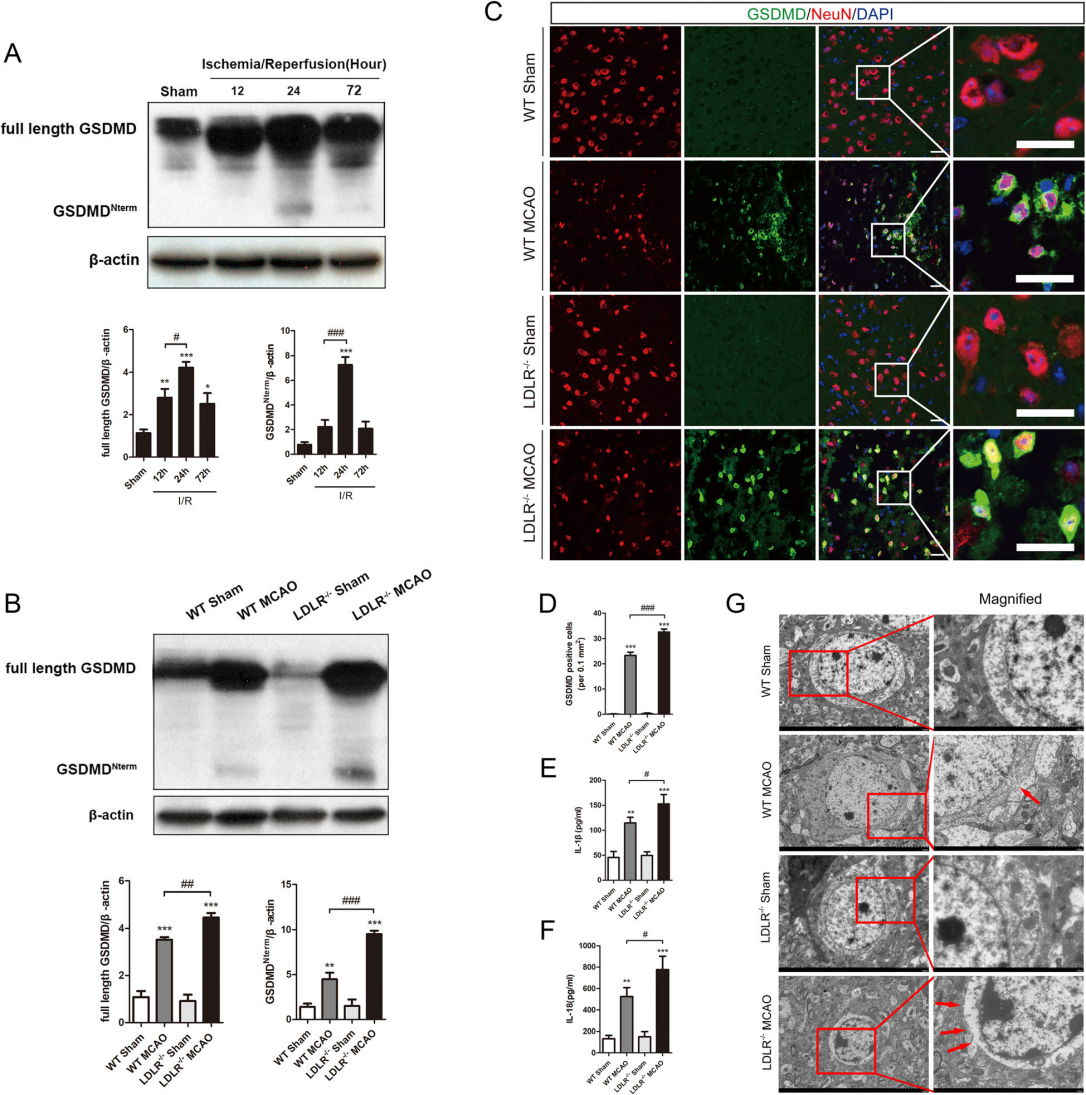
LDLR deficiency promotes neuronal pyroptosis in cerebral I/R injury. **a** Immunoblotting analysis for full-length GSDMD and N-terminal GSDMD in ischemic penumbra at 12, 24, and 72 h after MCAO, *n* = 5 for all groups. **b** Western blot and quantitative analysis of full-length GSDMD and N-terminal GSDMD 24 h after reperfusion, *n* = 6 in each group. **c**, **d** Double immunostaining of GSDMD with NeuN in peri-infarct region and quantitative analysis 24 h after reperfusion, *n* = 4–5. Insets show a higher magnification view. Scale bar = 20 μm. **e**, **f** ELISA for IL-1β and IL-18 levels in brain tissues, *n* = 5 in each group. **g** Representative transmission electron microscopy pictures of neurons in ischemic penumbra. Amplifies images of cytomembrane are labeled by red boxes. Pores on neuronal membrane are pointed out with red arrows, *n* = 3. Insets show a higher magnification view. Scale bar = 5 μm for the left images and 2 μm for the right images. Data are expressed as mean ± SEM. **P < 0.01, ***P < 0.001 versus WT sham mice; ^#^*P* < 0.05, ^##^*P* < 0.01, ^###^*P* < 0.001 versus WT MCAO mice

Previous studies have identified cerebral I/R-induced fierce cell pyroptosis in the ipsilateral ischemic hemisphere, where GSDMD may serve as a pivotal executioner [7, 10]. Therefore, we subsequently sought to determine whether LDLR could affect neuronal pyroptosis following ischemia. The western blot results showed that the expression levels of the full-length and N-terminal parts of GSDMD were both elevated at 12 h after reperfusion and reached a peak at 24 h after reperfusion, which remained at levels higher than baseline at 72 h after reperfusion (Fig. 4a, compared with Sham group, full-length GSDMD: *P* = 0.005 for reperfusion 12 h, *P* < 0.001 for reperfusion 24 h, *P* = 0.017 for reperfusion 72 h; N-terminal GSDMD: *P* < 0.001 for reperfusion 24 h). Cerebral I/R attack increased the expression of full-length GSDMD and N-terminal GSDMD to 3.3-fold and 3.2-fold higher than those in the sham group, which were both boosted by LDLR deletion (Fig. 4b, compared with the WT MCAO group, *P* = 0.006 for full-length GSDMD; *P* < 0.001 for N-terminal GSDMD). Immunostaining manifested that GSDMD-positive neurons were markedly increased in the ischemic penumbra region 24 h after MCAO, and the trends were notably enhanced by *Ldlr* knockout (Fig. 4c, d, compared with sham-operated group, *P* < 0.001 for WT MCAO group; compared with WT MCAO mice, *P* < 0.001 for the *Ldlr*^−/−^ MCAO group). Meanwhile, the secretion of IL-1β and IL-18 in ischemic penumbra was upregulated to 112.62 ± 12.67 pg/ml and 524.56 ± 83.96 pg/ml, which was further aggravated to 152.85 ± 18.66 pg/ml and 776.95 ± 124.74 pg/ml by LDLR deficiency (Fig. 4e, f, compared with WT MCAO group, *P* = 0.047 for IL-1β, *P* = 0.041 for IL-18). In addition, TEM images showed that more GSDMD pores were exhibited on neurons in *Ldlr*^−/−^ MCAO mice compared with those in WT MCAO mice (Fig. 4g).

**Fig. 5:**
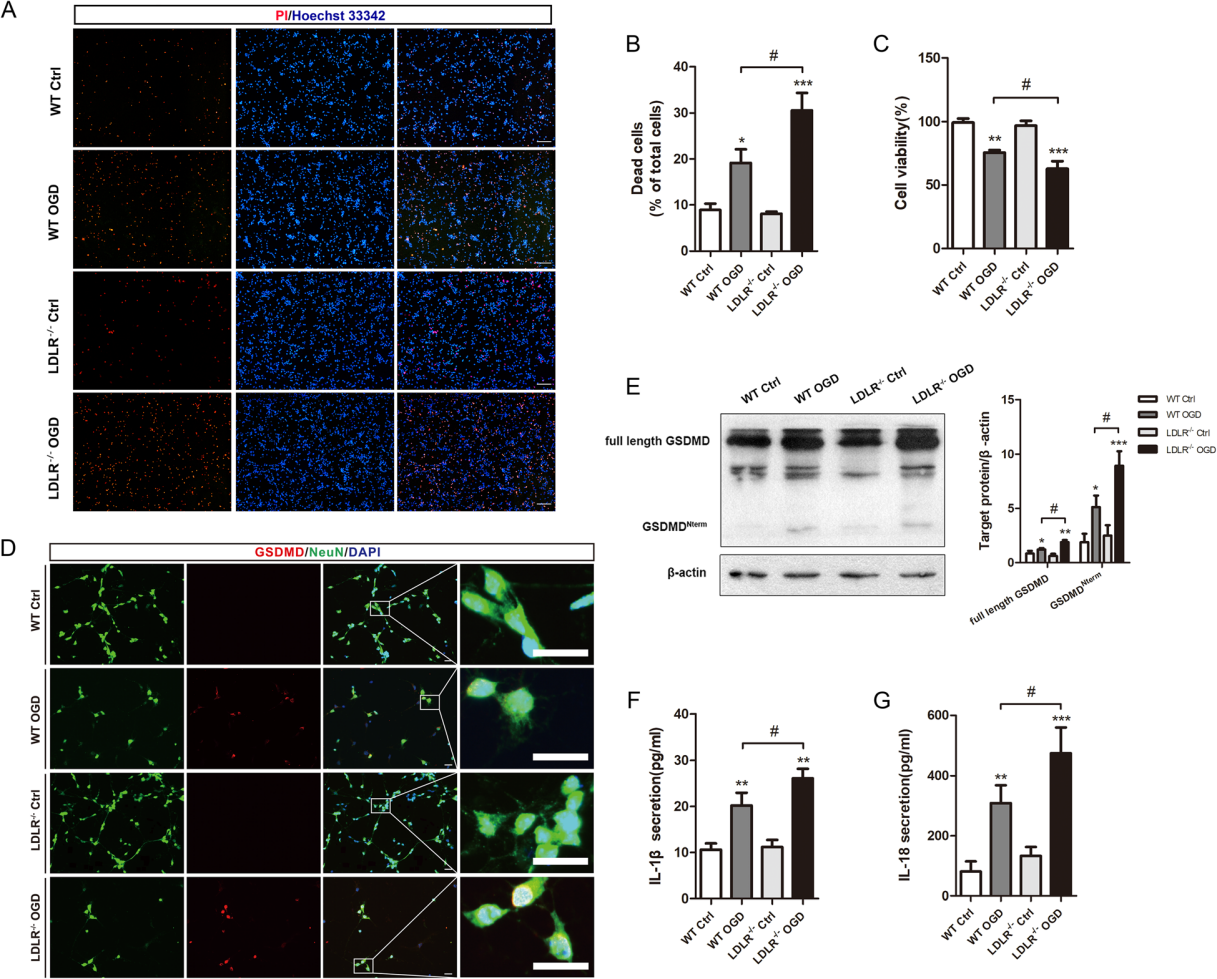
*Ldlr* deletion motivates neuronal pyroptosis in vitro. **a**, **b** Dead neurons are detected by PI/Hoechst staining 24 h after reoxygenation, *n* = 3. Scale bar = 50 μm. **c** Cell viability of cultured neurons 1 day post-OGD, *n* = 5–6 in each group. **d** Immunofluorescence staining for GSDMD and NeuN in the indicated groups, *n* = 4 in each group. Insets show a higher magnification view. Scale bar = 20 μm. **e** Western blotting analysis of full-length GSDMD and GSDMD Nterm in neurons 24 h after reoxygenation, *n* = 5. **f**, **g** Extracellular releases of IL-1β and IL-18 in OGD-treated neurons, *n* = 6–8. All results are expressed as mean ± SEM. *P < 0.05, **P < 0.01, ***P < 0.001 versus WT control neurons; ^#^*P* < 0.05 versus WT OGD neurons

To investigate the specific role of LDLR in neuronal pyroptosis, we cultured primary cortical neurons and performed OGD treatment. Neuronal death was provoked 24 h after OGD/R administration and was further magnified by LDLR elimination, as detected by PI/Hoechst staining (Fig. 5a, b, compared with WT OGD group, *P* = 0.013). Likewise, cell viability declined considerably in *Ldlr*^−/−^ neurons compared to that of WT neurons (Fig. 5c, *P* = 0.046). Immunofluorescence staining revealed that LDLR deletion further increased the number of GSDMD-positive neurons (Fig. 5d). The protein level of full-length GSDMD was augmented by OGD/R administration (Fig. 5e, compared with WT Ctrl group, *P* = 0.049 for WT OGD group) and more prominently under LDLR deficiency (Fig. 5e, compared with the WT OGD group, *P* = 0.04 for the *Ldlr*^−/−^ OGD group). An analogous tendency was found for the level of the N-terminal GSDMD in neurons, which was the key biological marker of pyroptosis (Fig. 5e, compared to WT OGD group, *P* = 0.021). As shown in Fig. 5f and g, the reoxygenation-induced release of IL-1β and IL-18 was also elevated in the culture medium of *Ldlr*^−/−^ OGD neurons (compared to WT OGD group, *P* = 0.041 for IL-1β; *P* = 0.048 for IL-18).

### *Ldlr* deletion amplifies NLRP3 inflammasome activation in cerebral I/R injury

With normal chow, plasma cholesterol levels were only 1.1-fold higher in *Ldlr*^−/−^ mice than levels in WT mice (Fig. S2A, *P* < 0.001). Since excessive cholesterol may affect sterile inflammation following ischemic stroke, tissue cholesterol levels were measured in different regions of the mouse brain. No significant differences were noted among the groups (Fig. S2B).

**Fig. 6:**
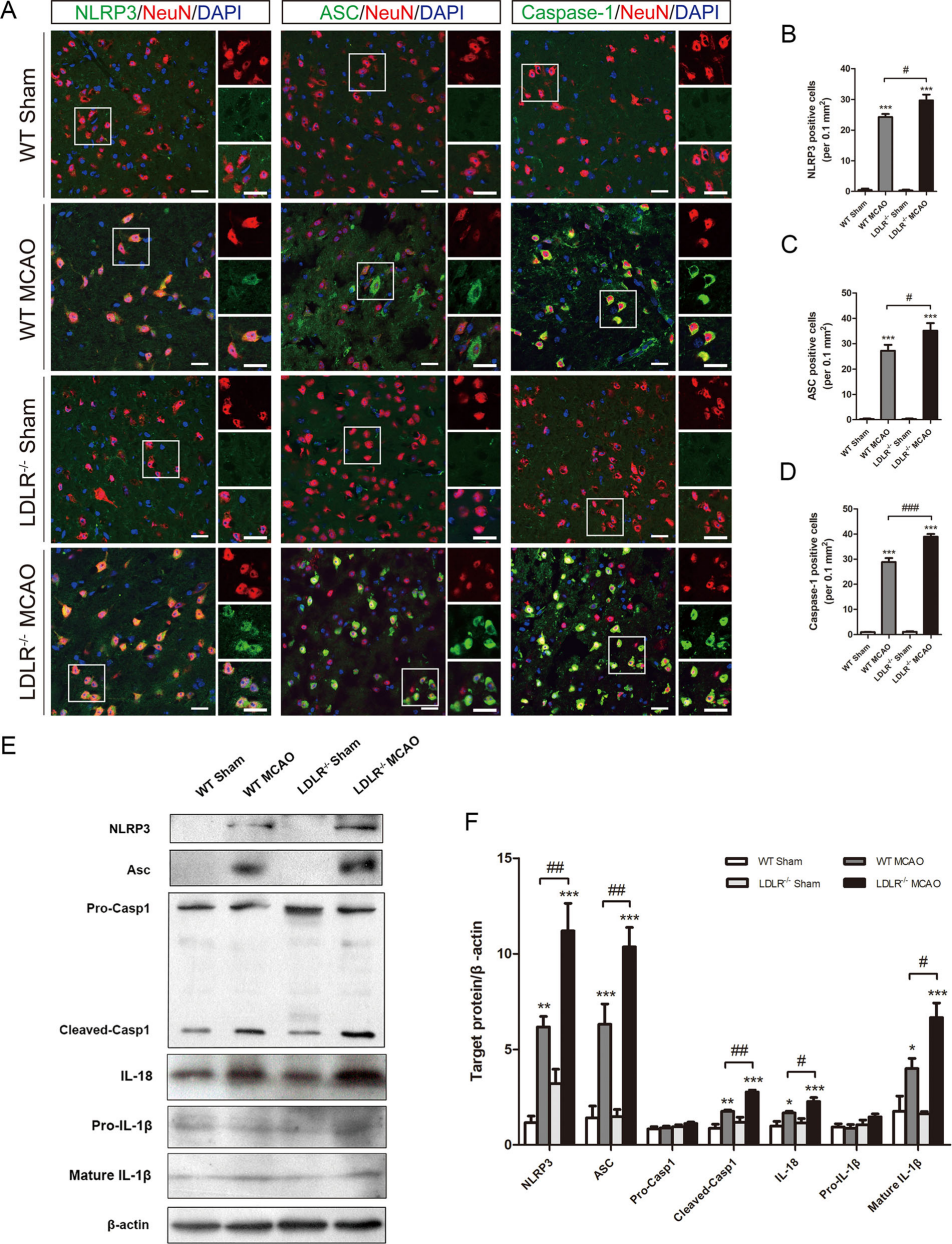
*Ldlr* knockout amplifies ischemia-induced NLRP3 inflammasome activation. **a**–**d** Representative immunofluorescence images of NLRP3, ASC, and caspase-1 were co-stained with NeuN in peri-infarct areas and their quantification 24 h after reperfusion, *n* = 3–5 in each group. Insets show a higher magnification view. Scale bar = 20 μm. **e**, **f** Western blotting and quantitative analysis of NLRP3, ASC, caspase-1, IL-18, and IL-1β expression in ischemic penumbra tissue 24 h after reperfusion, *n* = 4–6 for each group. Data are indicated as mean ± SEM. *P < 0.05, **P < 0.01, ***P < 0.001 versus WT sham mice; ^#^*P* < 0.05, ^##^*P* < 0.01, ^###^*P* < 0.001 versus WT MCAO mice. Casp 1, Caspase-1

The canonical pathway of pyroptosis is mediated by inflammasome activation following ischemic stroke, which also processes the precursors of IL-1β/18 into their mature forms and triggers neuroinflammation [27]. Given that the activation of the NLRP3 inflammasome requires an initial priming signal to evoke NF-κB signaling [2], we next asked whether LDLR could affect inflammasome activation and whether the NF-κB pathway was involved. Double staining results showed that ischemia-induced elevations of NLRP3, ASC, and caspase-1 were prominently augmented by LDLR deletion in peri-infarct regions (Fig. 6a–d, compared to WT MCAO group, *P* = 0.048 for NLRP3; *P* = 0.012 for ASC; *P* < 0.001 for caspase-1). Enhanced protein expression of NLRP3, ASC, cleaved caspase-1, mature IL-1β, and IL-18 was detected in *Ldlr*^−/−^ mice after I/R insult (Fig. 6e, f, compared to WT MCAO group, *P* = 0.001, 0.002, 0.001, 0.048, and 0.01, respectively). No significant differences were observed in the expression of caspase-1 precursor and pro-IL-1β (Fig. 6e, f).

**Fig. 7:**
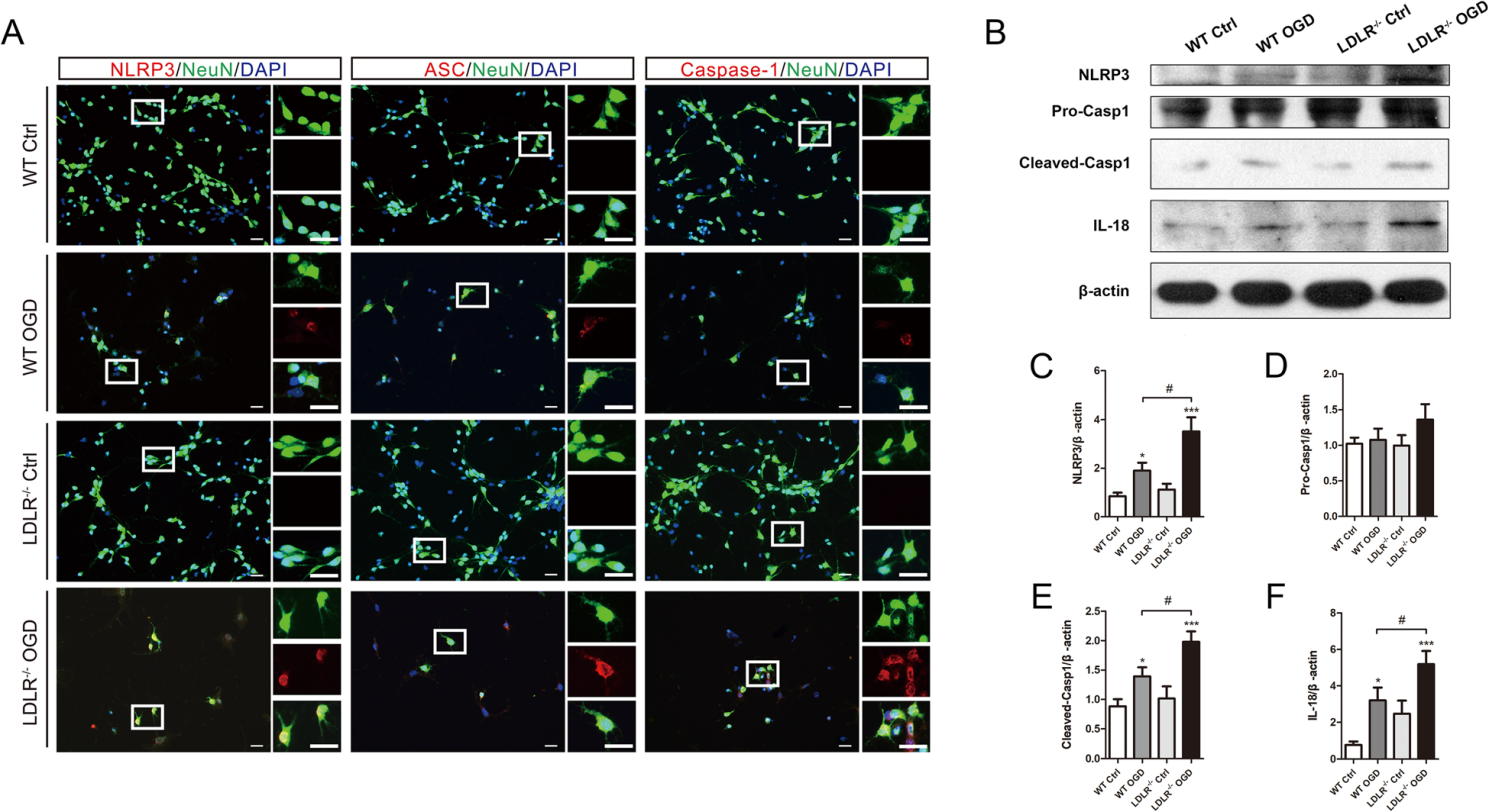
Absence of LDLR fortifies neuronal inflammasome activation after OGD/R. **a** Representative immunofluorescence images and quantitative analysis of NLRP3, ASC, and caspase-1 in cultured neurons 24 h after reoxygenation, *n* = 4. Insets show a higher magnification view. Scale bar = 20 μm. **b**–**f** Immunoblotting analysis and quantification of NLRP3, caspase-1, and IL-18 expressions in primary neurons, *n* = 5 for IL-18 and *n* = 4 for other proteins. All data are expressed as mean ± SEM. *P < 0.05, ***P < 0.001 versus WT control neurons; ^#^*P* < 0.05 versus WT OGD neurons

Consistently, as exhibited in Fig. 7a, in vitro OGD/R strengthened the immunofluorescence intensity of NLRP3, ASC, and caspase-1 in primary cultured neurons, which was further enhanced in *Ldlr*^−/−^ neurons. Deficiency of LDLR led to marked increases in the protein expression of NLRP3, cleaved caspase-1, and IL-18 in OGD/R neurons (Fig. 7b, c, e, f, compared with the WT OGD group, *P* = 0.016, 0.024, and 0.047, respectively). No significant difference was observed in the expression of caspase-1 precursor in vitro (Fig. 7b, d).

**Fig. 8:**
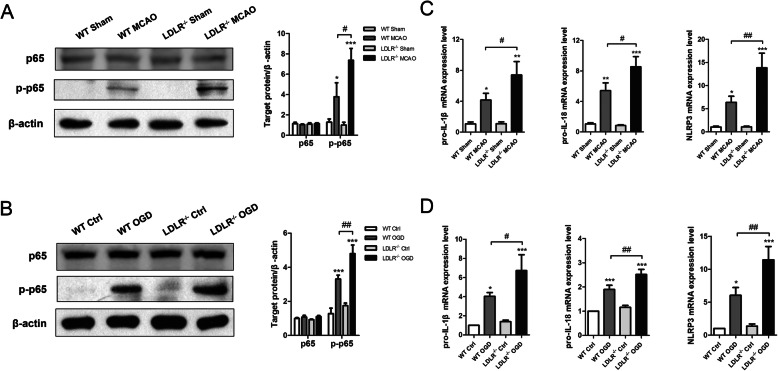
LDLR inhibition promotes NF-κB signaling activation. **a**, **b** Immunoblotting and quantitative analysis for p65, p-p65 in cerebral penumbra tissues, and cultured neurons, *n* = 4 for brain tissues and *n* = 3 for cultured neurons. **c**, **d** Real-time PCR for production of pro-IL-1β, pro-IL-18, and NLRP3 both in vivo and in vitro, *n* = 4–5. Data are shown as mean ± SEM. *P < 0.05, **P < 0.01, ***P < 0.001 versus WT sham or WT control group; ^#^*P* < 0.05, ^##^*P* < 0.01 versus WT MCAO or WT OGD group

Thereafter, the role of NF-κB pathway in NLRP3 inflammasome activation was detected. The protein levels of phosphorylated-p65 (p-p65) were upregulated by 3.6-fold in the penumbra regions of MCAO mice (Fig. 8a, compared to WT sham group, *P* = 0.019), while *Ldlr* knockout substantially increased their expression to 5.76-fold, respectively (Fig. 8a, compared to WT MCAO group, *P* = 0.043). Similar elevations of p-p65 expression were verified in cultured *Ldlr*^−/−^ neurons treated with OGD/R (Fig. 8b, compared to WT OGD group, *P* = 0.001). No significant difference was detected in the expression of p65 both in vivo and in vitro. A striking increase in pro-IL-1β, pro-IL-18, and NLRP3 mRNAs was confirmed in *Ldlr*^−/−^ mice following I/R examined by real-time PCR (Fig. 8c, compared with WT MCAO group, *P* = 0.038, 0.022, and 0.009, respectively). The mRNA levels of pro-IL-1β, pro-IL-18, and NLRP3 were also increased in *Ldlr*^−/−^ neurons after reoxygenation (Fig. 8d, compared to WT OGD neurons, *P* = 0.038, 0.007, and 0.007, respectively).

### Inhibition of NLRP3 reverses *Ldlr* deficiency-induced augment of neuronal pyroptosis following ischemia

**Fig. 9:**
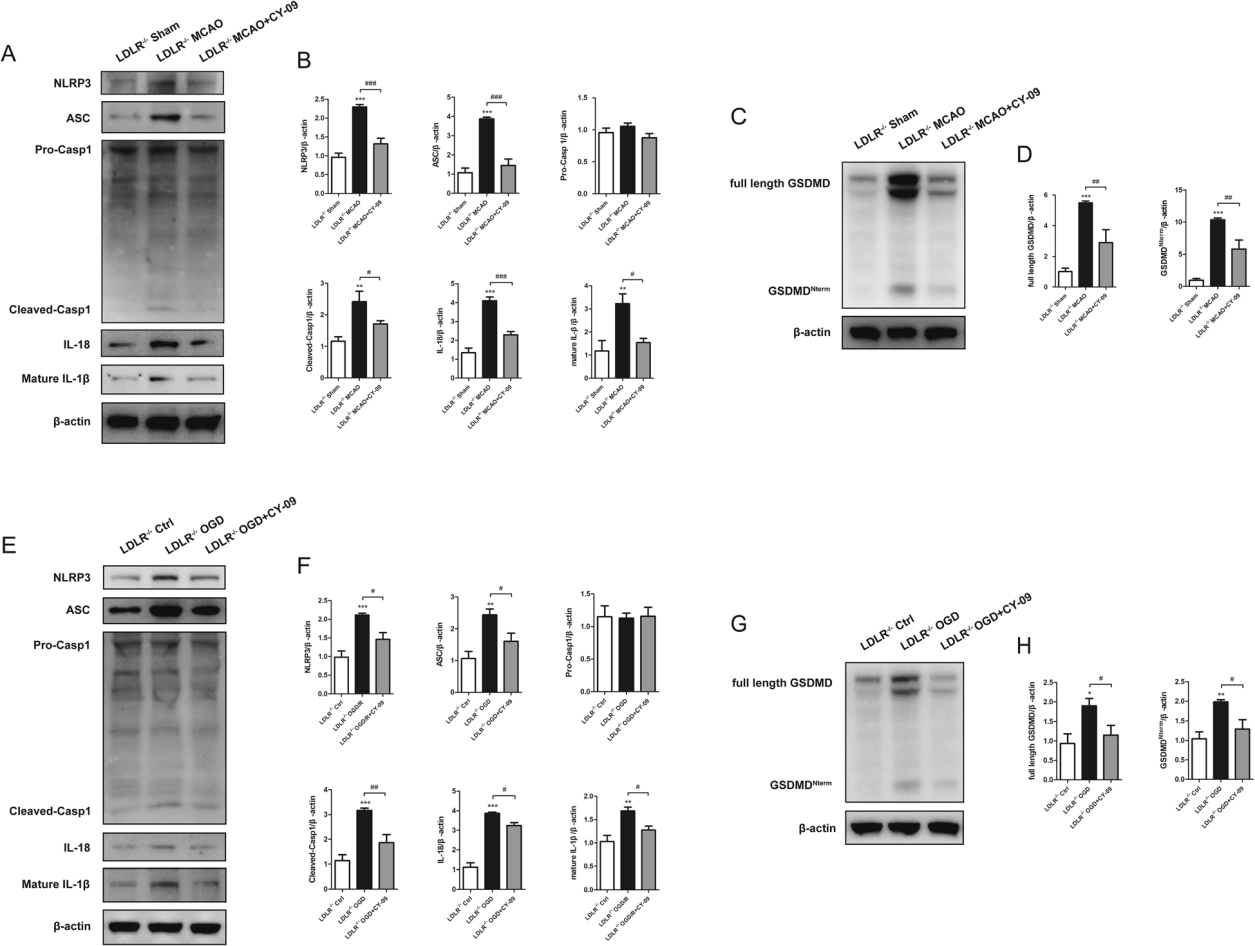
NLRP3 suppression retards *Ldlr* deletion-induced augment of neuronal pyroptosis. *Ldlr*^−/−^ MCAO mice and *Ldlr*^−/−^ OGD neurons were treated with NLRP3 inhibitor, CY-09. **a**, **b** Expressions of NLRP3, ASC, caspase-1, IL-18, and mature IL-1β in peri-infarct region were analyzed by immunoblots 24 h after ischemia with or without CY-09 injection. **c**, **d** Western blotting and quantitative analysis for GSDMD. **e**, **f** Immunoblotting analysis and quantitation for NLRP3, ASC, caspase-1, IL-18, and mature IL-1β in *Ldlr*^−/−^ neurons 24 h after OGD/R with or without CY-09 treatment. **g**, **h** Immunoblots and quantitative analysis for GSDMD in cultured neurons after OGD. *n* = 4 in each group. Data are shown as mean ± SEM. *P < 0.05, **P < 0.01, ***P < 0.001 versus *Ldlr*^−/−^ sham or *Ldlr*^−/−^ control group; ^#^*P* < 0.05, ^##^*P* < 0.01, ^###^*P* < 0.001 versus *Ldlr*^−/−^ MCAO or *Ldlr*^−/−^ OGD group

To investigate whether LDLR regulated neuronal pyroptosis and inflammatory response via mediating NLRP3 inflammasome, we treated *Ldlr*^−/−^ mice or *Ldlr*^−/−^ neurons with CY-09 to inhibit NLRP3. As shown in Fig. 9a and b, ischemia-triggered elevation of NLRP3 expression was conspicuously restrained with the administration of CY-09 (compared to *Ldlr*^−/−^ MCAO group, *P* < 0.001). The noticeable increment of ASC, cleaved caspase-1, mature IL-1β, and IL-18 levels in *Ldlr*^−/−^ mice were all suppressed by CY-09 (Fig. 9a, b, compared to *Ldlr*^−/−^ MCAO group, *P* < 0.001 for ASC, *P* = 0.045 for cleaved caspase-1; *P* < 0.001 for IL-18; *P* = 0.01 for mature IL-1β). Then, we detected the expression of GSDMD after NLPR3 inhibition. Employment of CY-09 significantly retarded LDLR deficiency-induced increment of full-length GSDMD and N-terminal GSDMD following ischemia (Fig. 9c, d, compared with *Ldlr*^−/−^ MCAO group, *P* = 0.005 for full-length GSDMD; *P* = 0.004 for N-terminal GSDMD). We next applied CY-09 to *Ldlr*^−/−^ neurons. The elevated expression of NLRP3 induced by OGD was refrained by CY-09 (Fig. 9e, f, compared to *Ldlr*^−/−^ OGD group, *P* = 0.012). Similar tendencies of ASC, cleave caspase-1, IL-18, IL-1β, full-length GSDMD, and N-terminal GSDMD expressions were also observed in *Ldlr*^−/−^ neurons treated with CY-09 after OGD/R (Fig. 9e–h, compared with *Ldlr*^−/−^ OGD group, *P* = 0.027, 0.004, 0.021, 0.018, 0.048, 0.021, respectively). All these results demonstrated that LDLR regulates neuronal pyroptosis and neuroinflammation via NLRP3 inflammasome pathway.

## Discussion

**Fig. 10:**
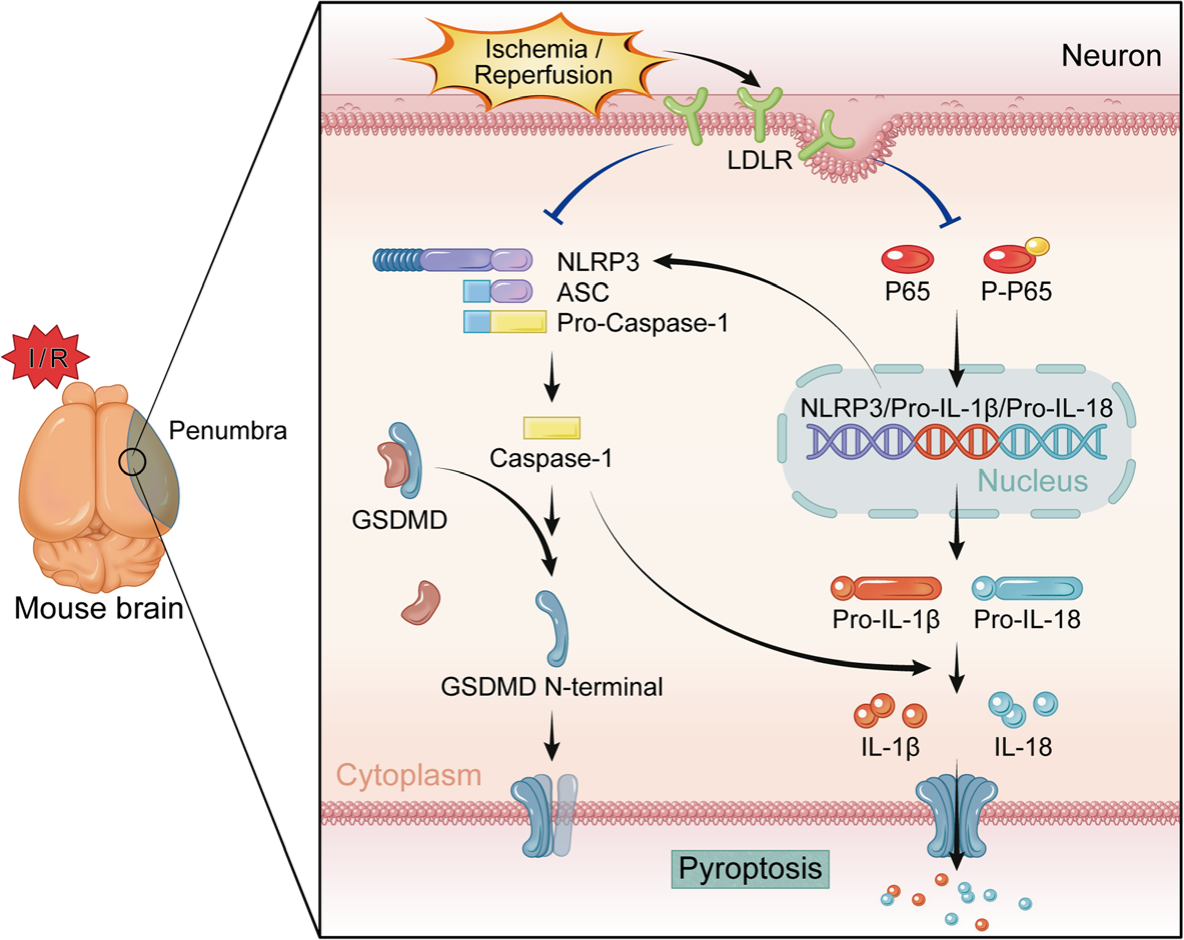
Schematic diagram for the mechanisms of LDLR in the regulation of neuronal pyroptosis following cerebral ischemia. The expression of LDLR is downregulated after Cerebral I/R. LDLR restrains two-step processing pathways, including NF-κB signaling (priming) and NLRP3-ASC-caspase-1 inflammasome assembly, to mitigate generating inflammatory mediators and cytokines. The triggered caspase-1 cleaves GSDMD to promote the release of N-terminal domain, which executes pores formation on neuronal membrane. The mature forms of IL-18 and IL-1β secreted through these pores are also alleviated, which facilitates anti-inflammatory effect post-stroke

The present study demonstrated that the expression of LDLR was downregulated following acute cerebral ischemia. Furthermore, *Ldlr* genetic knockout exacerbated neuronal pyroptosis and inflammatory response by provoking NLRP3 inflammasome activation and recruitment, leading to cerebral infarct volume enlargement and neurological deficit aggravation (Fig. 10). Inhibition of NLRP3 could reverse enhanced neuronal pyroptosis induced by *Ldlr* deficiency after ischemia. Moreover, post-stroke long-term cognitive and memory impairments of mice were deteriorated by *Ldlr* deletion.

Neuroinflammation in the CNS is an essential process in the pathophysiology of ischemic stroke, which can result in severe nerve injury and long-term neurobehavioral dysfunction [28]. However, the progress in seeking proper treatments aimed at reducing neuroinflammation following ischemia remains limited. Previous evidence has provided insight into a sterile inflammatory mechanism known as “inflammasome” during acute cerebral ischemia [29]. Inflammasomes are innate immunity hubs, generally composed of a stimulus-detecting sensor, the adaptor molecule ASC, and the protease precursor pro-caspase-1, playing a critical role in the initiation of innate immune response through sensing stimulus-induced danger-associated molecular patterns (DAMPs) released from infarct regions [30–34]. Among multiple inflammasome-forming proteins, NLRP3 has been reported to regulate neuroinflammation and neuronal death in ischemic stroke [1, 32, 35, 36]. Recognition of DAMPs mediated by surface PPRs subsequently triggers enhanced downstream transcriptional activities of pro-IL-1β, pro-IL-18, and NLRP3 via nuclear factor kappa B (NF-κB) in an autocrine or paracrine manner, which serves as signal 1 (priming) of the inflammatory response [34]. After NLRP3 binding with the adaptor protein ASC, pro-caspase-1 is converted into its biologically active form and subsequently triggers the maturation of precursor IL-1β and IL-18 [37, 38]. In addition, the NLRP3 inflammasome evokes proinflammatory GSDMD-executed pyroptotic cell death [39, 40]. As the key protein in pyroptosis, GSDMD belongs to a gasdermin family, including GSDMA, GSDMB, GSDMDC, GSDMD, GSDME, and DFNB59 [41, 42]. Evidence has shown that full-length GSDMD contains a 31-kDa N-terminal fragment and a 22-kDa C-terminal fragment, in which the C-terminus exerts an auto-inhibitory function in the resting state [43]. Upon cleaved at the D276 cleavage site by caspase-1, the N-terminus of GSDMD specifically anchors to cellular membrane lipids and oligomerizes to form permeability pores whose inner diameter is 10–14 nm [44, 45]. Thereafter, these pores dissipate cellular ionic gradients, which then cause water influx, cell swelling, eventual osmotic lysis, and release of inflammatory intracellular contents [12]. Pyroptosis has been demonstrated to occupy a crucial place in nerve cell death after cerebral ischemic injury [7, 10, 46]. Analogously, our study observed an upregulated expression of full-length GSDMD and its N-terminus cleavage product via NF-κB signal-primed inflammasome cascades in the acute phase of cerebral ischemia. A similar increase of N-terminal region oligomer-forming pores on the membranes of neurons was also revealed.

As an endocytic transmembrane receptor localized on the plasma membrane, LDLR can combine and take up extracellular ligands such as low-density lipoprotein and apolipoprotein E, playing a crucial regulatory role in lipid and cholesterol metabolism [47]. LDLR contains a cluster of seven ligand-binding repeats, an EGF homology region and a sugar domain in the extracellular domain, and an NPxY motif in the cytoplasmic domain mediating ligand endocytosis and signal transduction via coated pits [48]. The regulation of LDLR is implemented through sterol regulatory element binding proteins (SREBPs) and secreted proprotein convertase subtilisin-like/kexin type 9 (PCSK9) at the transcriptional and posttranscriptional levels, respectively [49]. It has been confirmed that chronic inflammation could disrupt the LDLR pathway, giving rise to lipid disorders in atherosclerosis, nonalcoholic fatty liver disease, diabetes, and chronic kidney disease [14, 50, 51], while emerging studies have shown that LDLR in turn plays a vital role in inflammatory reactions. The overexpression of LDLR via reducing PCSK9 function was associated with a decreased inflammatory cytokine response and improved septic shock outcomes in both mice and humans [52]. Likewise, it was observed that PCSK9 fortified atherosclerotic inflammation and cell apoptosis in an LDLR-dependent mechanism [53]. In the brain, neuroinflammation of nerve cells was also shown to be mitigated in *Ldlr* transgenic mice with Alzheimer’s disease [54], implying that LDLR may be involved in mediating the sterile inflammatory process of brain tissues. In this investigation, we found that *Ldlr* knockout pronouncedly exacerbated the risk of cerebral I/R-induced neuronal pyroptosis. The activation of the NLRP3 inflammasome in *Ldlr*^−/−^ mice was reinforced through the NF-κB signaling pathway following acute ischemia. In addition, inhibition of NLRP3 could reverse *Ldlr* deficiency-induced augment of neuronal inflammation and pyroptosis. These data indicated that LDLR could regulate NLRP3-mediated neuronal pyroptosis after cerebral I/R, implying the protective role of LDLR as an inflammatory mediator in ischemic stroke. Consistent with our findings, a study has shown that *Ldlr* knockout augmented inflammatory cell infiltration in mouse aortas, although the underlying mechanism remains to be elucidated [55]. Nevertheless, our data conflict with a report that the increase in LDLR expression did not protect mice from LPS-induced death [56]. The contradictory roles of LDLR in the regulation of cell survival may be ascribed to the differences in cell types, experimental settings, and stimulus approaches.

Genetic ablation of *Ldlr* has been established to disrupt cholesterol homeostasis and cause the onset of atherosclerosis, such as familial hypercholesterolemia [16]. *Ldlr* deletion was verified to instigate the amplification of the inflammatory response in macrophages and other immune cells [57]. Hence, we assessed whether LDLR deletion would raise the level of cholesterol in brain tissue and consequently influences the inflammation motivation of nerve cells. Total cholesterol levels in the cortex, hippocampus, corpus callosum, and cerebellum were examined in our study, and the results showed no significant difference between WT and *Ldlr*^−/−^ mice. Therefore, it may rule out the impact of cerebral cholesterol changes on inflammatory responses in the brain. It has been reported that mitochondria isolated from low-fat-fed *Ldlr*^−/−^ mice tissues produced more ROS, while attenuated LDLR degradation could cause a decrease in ROS generation [58, 59]. Another research also observed that *Ldlr* mutant macrophages presented increased levels of oxidants and inflammatory cytokines [60]. Simultaneously, experimental evidences demonstrated that the increase in ROS may serve as a triggering factor to activate NLRP3 inflammasome [61–63]. Subsequent study has indicated that the elimination of ROS alleviated the cleavage of GSDMD and pyroptosis [64]; hence, further investigations are required to determine the potential mechanism of LDLR regulating ischemia-induced neuronal pyroptosis. Shc (p66) protein is a cellular signaling adaptor transducing signals of transmembrane proteins [65, 66]. Previous studies have demonstrated that Shc strongly promotes cellular ROS generation [67–69]. It is possible that LDLR may act on the ROS signaling pathway to regulate inflammasome activation downstream via interacting with the Shc protein.

## Conclusions

In conclusion, this study demonstrated that LDLR regulated neuronal pyroptosis induced by cerebral I/R, which may exert a protective effect on neurons and improve neurological dysfunction following cerebral ischemia. To our knowledge, these results have for the first time disclosed the correlation of LDLR with NLRP3-induced neuronal pyroptosis post-stroke. Our findings highlighted a crucial role of LDLR in the suppression of neuroinflammation and may represent a therapeutic target in the treatment of inflammasome-associated diseases.

## Supplementary information

**Additional file 1: Fig. S1.** The location of LDLR protein in various brain regions. (A,B) Double staining images of LDLR with NeuN were pictured in corpus callosum and hippocampus, *n* = 3. Scale bar = 20μm. CC, Corpus callosum.

**Additional file 2: Fig. S2.** Cholesterol levels in the plasma and different brain tissues of mice. (A) Cholesterol level in mice plasma. (B) Total cholesterol level in various brain areas. *n*= 4 for all groups. Data are expressed as mean ± SEM. ^***^P<0.001 versus WT mice.

## Data Availability

All data generated or analyzed during this study are included in this published article and its supplementary files.

## Abbreviations

ASC: Apoptosis-associated speck-like protein containing a caspase recruitment domain
Caspase-1: Cysteinyl aspartate specific proteinase-1
DAMPs: Danger-associated molecular patterns
DAPI: 4′,6-diamidino-2-phenylindole
GSDMD: Gasdermin D
IL-1β: Interleukin-1β
IL-18: Interleukin-18
I/R: Ischemia/reperfusion
LDLR: Low-density lipoprotein receptor
MCAO: Middle cerebral artery occlusion
NLRP3: Nucleotide-binding oligomerization domain (Nod)-like receptor pyrin domain containing 3
OGD: Oxygen-glucose deprivation
PCSK9: Proprotein convertase subtilisin/kexin type 9
PRR: Pattern recognition receptor
SREBPs: Sterol regulatory element binding proteins
