# Supplementary figures and images for: Low-density lipoprotein receptor (LDLR) regulates NLRP3-mediated neuronal pyroptosis following cerebral ischemia/reperfusion injury
Source: J Neuroinflammation. 2020 Nov 5;17:330. doi: 10.1186/s12974-020-01988-x (PMC7643474; doi:10.1186/s12974-020-01988-x)

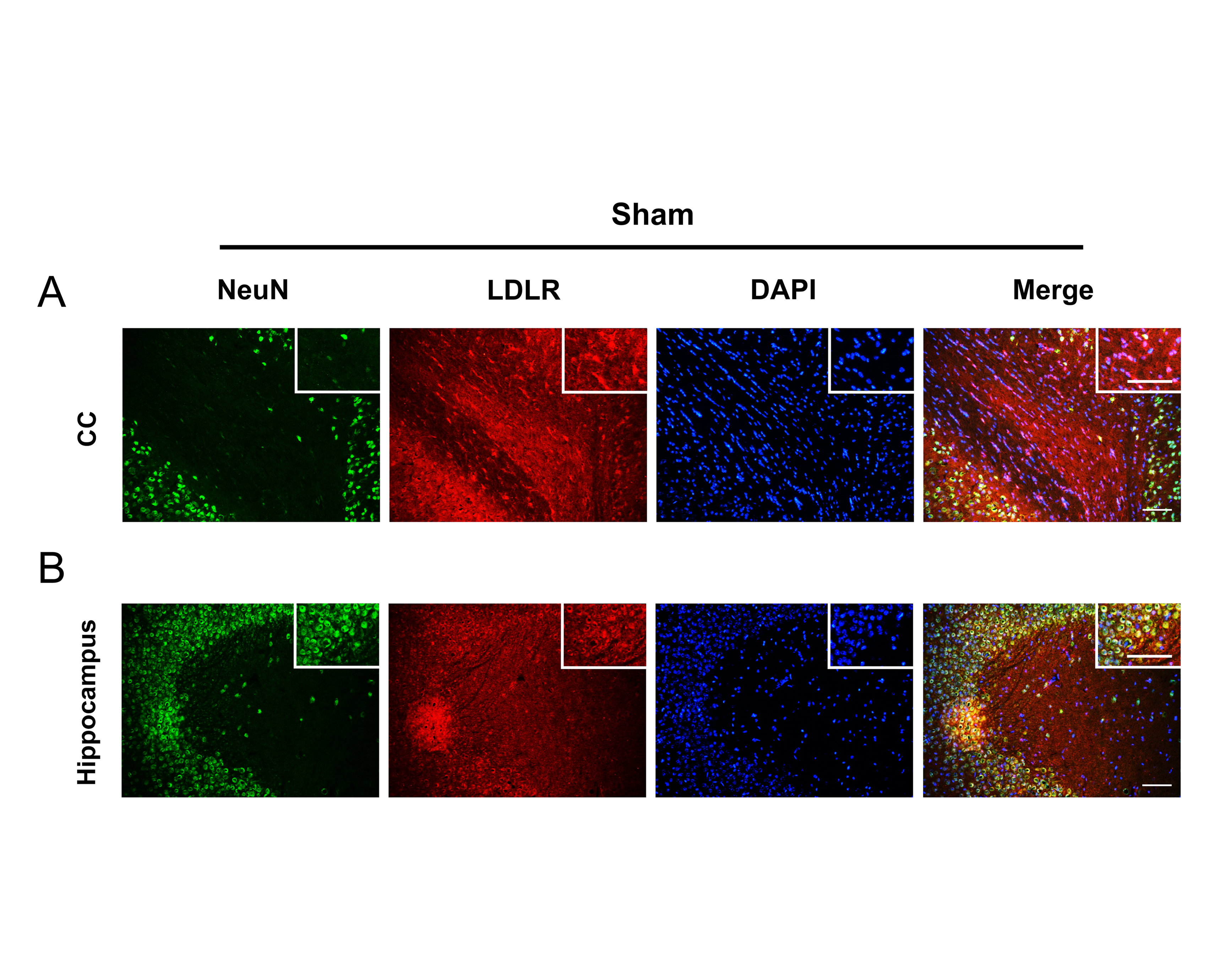

Supplement: Supplementary file 1 — Additional file 1: Fig. S1. The location of LDLR protein in various brain regions. (A,B) Double staining images of LDLR with NeuN were pictured in corpus callosum and hippocampus, n = 3. Scale bar = 20μm. CC, Corpus callosum. [file 12974_2020_1988_MOESM1_ESM.tif]

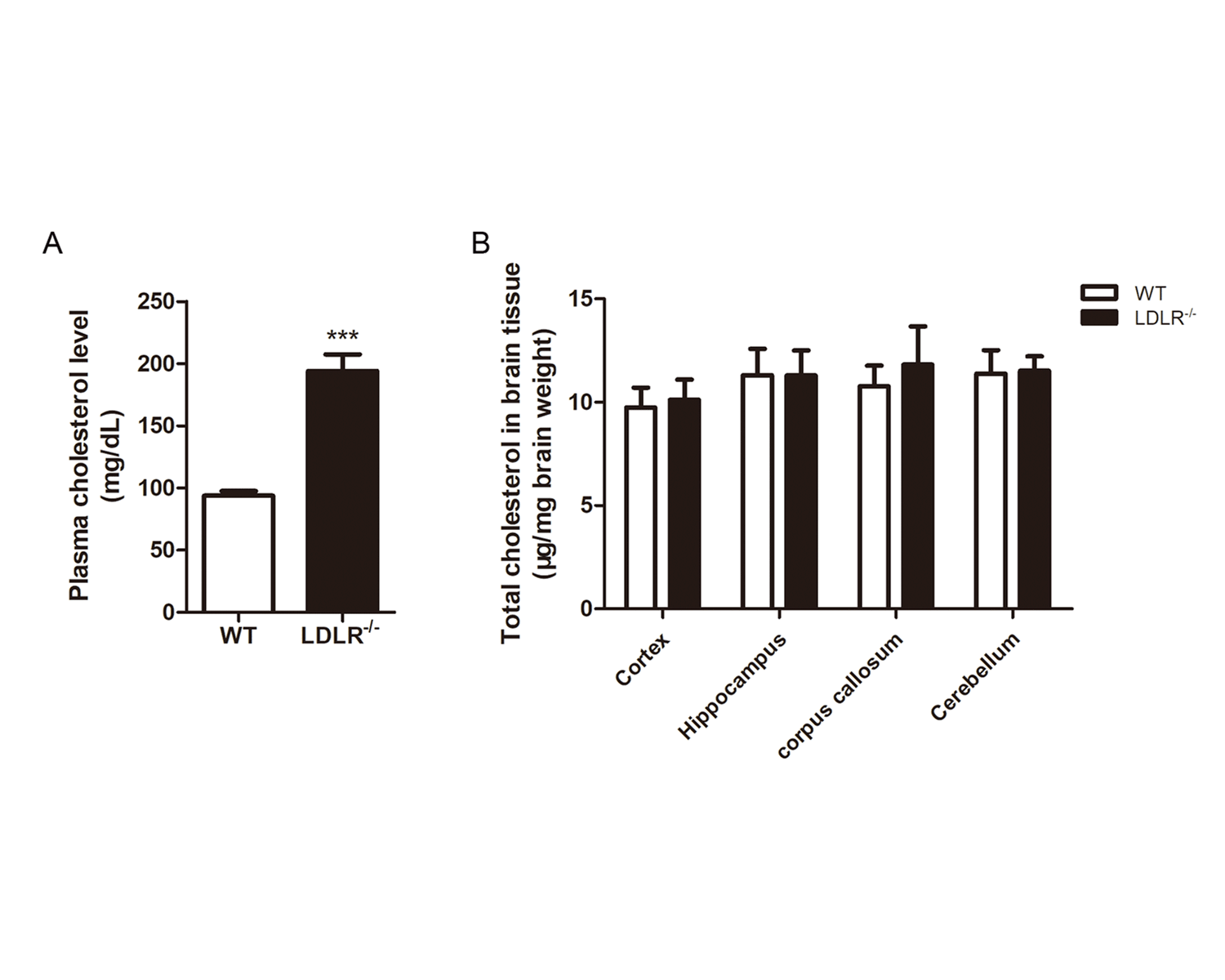

Supplement: Supplementary file 2 — Additional file 2: Fig. S2. Cholesterol levels in the plasma and different brain tissues of mice. (A) Cholesterol level in mice plasma. (B) Total cholesterol level in various brain areas. n= 4 for all groups. Data are expressed as mean ± SEM. ***P<0.001 versus WT mice. [file 12974_2020_1988_MOESM2_ESM.tiff]
